# Correlates of fertility desires in women with urogenital fistula in the Democratic Republic of Congo: a cross-sectional study of 1,646 women

**DOI:** 10.1186/s12978-024-01823-z

**Published:** 2024-07-03

**Authors:** Guerschom Mugisho-Munkwa, Raha Maroyi, Denis Mukwege

**Affiliations:** 1 Demography Institute, Paris 1 Panthéon-Sorbonne University, Paris, France; 2 Université Evangélique en Afrique (UEA), Bukavu, Democratic Republic of Congo; 3 Department of Obstetrics and Gynecology, Panzi Hospital, Bukavu, Democratic Republic of Congo; 4 Ecole des Hautes Etudes en Démographie (HED), Paris, France

**Keywords:** Fertility desires, Fertility, Fistula, Democratic Republic of Congo

## Abstract

**Background:**

Studies on fertility desires among fistula patients in the Democratic Republic of Congo (DRC) have been conducted on fewer patients. Furthermore, these studies have adopted a univariate descriptive approach. This study aims to examine the determinants of fertility desires among patients with fistula in the DRC.

**Methods:**

This cross-sectional study included women aged 15–49 whose fistulas were repaired by the Panzi Hospital mobile team in seven DRC regions between 2013 and 2018. Univariate and bivariate descriptive analyses were performed using the frequency distribution table and the chi-square test. Adjusted odds ratios with their 95 confidence intervals from logistic regression were used to analyze factors associated with fertility desire after fistula repair. All analyses were stratified by parity level for all women aged 15–49 and 20–34 years.

**Results:**

Of the 1,646 women aged 15–49 and 808 aged 20–34, 948 (57.6%) and 597 (73.8%), respectively, wanted to have children after fistula repair. Among women aged 15–49 and 20–34 years, the desire to have children was parity-specific. It was negatively associated with age at all parity levels. In women with low parity, the desire for children was significantly negatively associated with a high number of surgeries, abortions, and fistula duration. It tended to decrease with time, but was particularly high in 2014 and 2017. It was high among the Protestant women. Among medium-parity women, it was significantly lower in urban areas and among widows, but higher among women who had more than two abortions. Among high-parity women, it was negatively associated with education level.

**Conclusion:**

To help women with fistula achieve or approach their desired number of children, our findings suggest that (1) counseling is needed for women with a high desire for children; (2) the human, material, and financial resources needed to eliminate fistula in the DRC should be made available; and (3) medical and nursing staff should be sufficiently and effectively trained to minimize the number of unsuccessful surgeries performed on women with fistula.

**Supplementary Information:**

The online version contains supplementary material available at 10.1186/s12978-024-01823-z.

## Background

In 2006, 2 to 3.5 million women worldwide had obstetric fistula, and 30,000 to 130,000 additional cases occur annually due to poor delivery settings [1]. In 2007, 1.8% of women aged 15–49 in the Democratic Republic of the Congo (DRC) had obstetric fistula [2]. Young, impoverished, illiterate women in rural areas without health facilities are particularly at risk of fistula. Fistula is a prominent cause of child and maternal death and stillbirth in poor countries [3].

DRC social services are substandard, owing to the vastness of the country and the weak road network. Access to contraception remains poor (20.4% current contraceptive usage among married women and 27.7% unmet needs for family planning in 2013) [4]. In addition, the prevalence of sexual violence with extreme violence has led to an “epidemic of traumatic fistula” [5, 6]. In the DRC, about 42,000 women with fistula were waiting for surgical treatment in 2007 [4, 7]. Fistula remained one of the most neglected conditions [8] until 2008, when a special USAID prevention and surgical management program, Fistula Care Plus, was launched in 14 developing countries, including the DRC. The Fistula Care Plus program aims to reduce the prevalence of fistula and help women with obstetric or gynecologic trauma reintegrate into society.

Fistula-afflicted women may have distinct fertility desires than fistula-free women resulting from separation from their husbands and health issues [9]. According to Miller’s Traits-Desires-Intentions-Behavior (T-D-I-B) framework [10–12], childbearing motivations leads to fertility desires, fertility intentions and subsequent fertility. The first step leading to childbearing is the formation of motivations. These characteristics (such as health status, marital status, age, perceived value of children, parity) cause an individual to respond in a certain way in certain circumstances. The motivations are in turn activated as the individual’s desire for children (the wish to have more children or to remain childless), which is then translated into intentions to have children (more concrete plans to pursue childbearing). There is a strong correlation between desires and intentions, but they are conceptually distinct. Desires are representations of what people want to do, not necessarily what they plan to do [13, 14]. High fertility intentions are then translated into actual childbearing when opportunities arise. Studies show that fertility desires and fertility are very closed in Africa [15].

Existing research on fertility desires among fistula patients in the DRC were done on fewer participants and employed a univariate descriptive approach [16]. Thus, the factors associated with the desire to have one or more children among women with fistula remain unknown. Knowledge of these factors can help guide contraceptive counseling. Contraceptive education for women with or who have had a fistula is especially important, as pregnancy can occasionally trigger a recurrence of the fistula [16]. Previous research on women who became pregnant following fistula treatment has indicated that fistula recurred in 11% of women who gave birth after a satisfactorily healed fistula [17]. To help these women achieve their fertility goals without jeopardizing their own health, accurate information about their fertility desires and intentions is essential. From a demographic standpoint, the study of fertility desires and related factors in fistula patients, as well as fistula-free women, can help predict future fertility in women’s living environments [18]. Finally, knowledge of fertility desires will allow to predict contraceptive use among women with obstetric fistula [15].

This study aims to analyze factors associated with fertility desires among women with obstetric fistula.

## Methods

### Data and study population

This cross-sectional study was based on data collected from 2013 to 2018 as part of the Fistula Heath Care Program from women whose fistulas were repaired at Panzi Hospital in South Kivu Province and six other provinces in the DRC. The fistula repairs were performed by the Panzi Hospital’s mobile team and by doctors working at local hospitals that hosted the Panzi mobile team. The Panzi teams, consisting of at least two surgeons, a surgical assistant, a nurse, and an anesthesiologist at each site, are deployed annually or bi-annually, depending on the number of fistulas and the financial and logistical resources available, or at the request of the host sites through NGOs, local churches, civil society, or women’s associations. Once the site is identified, an agreement is signed that allows the Panzi Mobile Team to provide expertise in patient consultation, surgical equipment, medications, and quarterly patient follow-up. Each host site has at least one medical director to manage operations and teams, a nursing director to oversee pre- and post-operative care, and a religious or civil society leader to provide information to patients and their families and to oversee the nutrition of women whose fistulas have been repaired [19].

Data were collected using a ten-page form that included sociodemographic information, gynecologic and obstetric history (pregnancies, abortions, child survival status, current health status of the patient, surgical history and information on recent fertility, fertility aspirations, knowledge, attitudes and practices regarding fistula, diagnosis, treatment and outcome of surgery). Subsequently, the data were entered into a database designed on Epi-Info for this purpose.

### Outcome and variables

The outcome variable in this study was the woman’s desire to have another child after fistula repair. This variable was measured using the following question: ‘Do you want to have children after your medical treatment?’ Responses to this question were coded 1 (yes) if the woman wanted to have children after fistula repair and 0 (no) otherwise.

We tested the effect of sociodemographic variables including age (15–19, 20–24, 25–29, 30–34, 35–39, 40–44, 45–49 years), marital status (married, separated/divorced, widow), occupation (farmer, housekeeper, seller, other/unknown), religion (Protestant, Catholic, other), highest level of formal education (no formal education, primary school, secondary school), year of fistula repair (from 2013 to 2018), province (Equateur, Kasai-Oriental, Katanga, Province Orientale, and Kivu, which includes South Kivu, North Kivu, and Maniema, combined into Kivu due to small sample size), place of residence (urban or rural) and parity. Following other studies [20, 21] and to balance the sample size between categories, parity was coded as 0–2 children (low parity), 3–4 children (medium parity) and ≥ 5 children (high parity). We also tested the effect of variables related to women’s health: fistula duration (0–9, 10–19, ≥ 20 years), years since last delivery (0–4, 5–9 and ≥ 10), number of abortions (0, 1–2, ≥ 3), and number of surgeries before fistula repair (0, 1–2, ≥ 3). To maintain a large sample size, a separate category ‘Unknown/Not applicable’ was used for missing information on any variable.

### Statistical analysis and analysis strategy

Frequency and contingency tables were used to describe data. A chi-square test was used to test the relationship between each independent variable and the desire to have a child. Adjusted odds ratios along with their 95% confidence intervals (CI) from a binary logit model were used to analyze factors associated with the desire to have a child after fistula repair. We tested for collinearity between the explanatory variables using generalized variance inflation factors (GVIF). One variable (province) that was more collinear with other variables, GVIF > 3 (see Supplementary Table 1), was dropped from the final model. Statistical significance was set at *P* < 0.05. Analyses were performed using *lme4*, *car* and *gtsummary* packages in R.

We first conducted analyses on all fistula patients aged 15–49, as fertility analysis conventionally focuses on women of fertile age, 15–49 years [22]. Results for all women aged 15–49 may be useful, for example, for predicting fertility based on fertility desires or intentions. All analyses were stratified by parity for three reasons. First, parity is an important determinant of fertility desires [23]. Second, parity may be a confounding factor in that it is associated with other factors such as the number of surgeries or the number of abortions [24]. Third, certain authors suggest that childbearing decisions are made sequentially [24, 25]. This parity-specific design allowed us to investigate how child demand varies for each parity level.

We also conducted analyses on women aged 20–34 years. The 20–35 age group would be preferable, but the age variable was available in five age groups. There are four reasons for using this strategy. First, age is another major factor in fertility and fertility desires and may be correlated with other factors. Second, 20–35 is the most fertile age group. In other words, women tend to have more children in this age range. Third, childbearing is less risky in this age interval [26, 27]. Fourth, this age group may be more relevant for policy development; that is, policymakers can still help them achieve their desired family size without compromising their health.

## Findings

### Sample description and bivariate analysis of factors associated with fertility desires after fistula repair

**Fig. 1:**
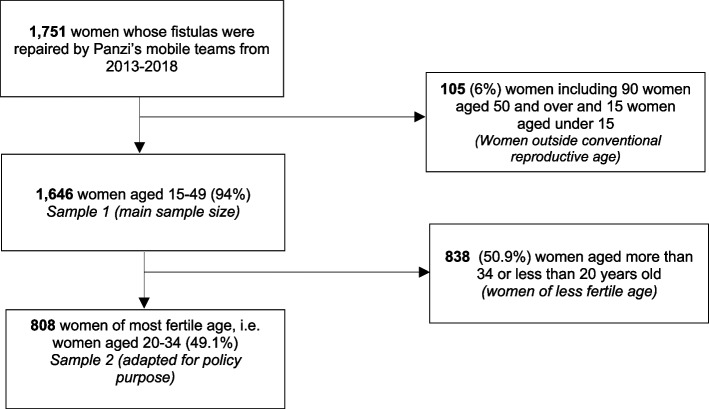
Flow chart of inclusion and exclusion criteria in the study population

The sample size consisted of 1,646 women. Figure 1 depicts the flowchart for the sample selection procedure.

Table 1 presents the descriptive statistics and bivariate analysis of factors associated with the desire to have children after fistula repair for all women aged 15–49 years (all parities combined). Table 2 presents the same information by parity level.

**Table 1:** Descriptive statistics and bivariate analysis of factors associated with the desire for children after fistula repair among all women aged 15–49

**Variables**	**Categories**	N (%)^1^	Number and % of women desiring to have children	*P*-value^2^
		**1,646 (100)**	948 (57.6%)	
**Age**	15–19	127 (7.7%)	109 (86%)	< 0.001***
	20–24	263 (16%)	228 (87%)	
	25–29	269 (16%)	202 (75%)	
	30–34	276 (17%)	167 (61%)	
	35–39	198 (12%)	123 (62%)	
	40–44	149 (9.1%)	53 (36%)	
	45–49	364 22%)	66 (18%)	
**Marital status**	Married	1,076 (65%)	637 (59%)	0.005***
	Separated/Divorced	302 (18%)	183 (61%)	
	Widow	136 (8.3%)	66 (49%)	
	Unknown	132 (8.0%)	62 (47%)	
**Profession**	Farmer	252 (15%)	162 (64%)	< 0.001***
	Housekeeper	1,123 (69%)	649 (58%)	
	Seller	50 (3.0%)	38 (76%)	
	Other/Unknown	221 (13%)	99 (45%)	
**Religion**	Catholic	476 (29%)	241 (51%)	< 0.001***
	Other	297 (18%)	136 (46%)	
	Protestant	873 (53%)	571 (65%)	
**Highest diploma**	Did not complete primary school	676 (41%)	368 (54%)	0.003***
	Completed primary school	642 (39%)	403 (63%)	
	Completed secondary school	328 (20%)	177 (54%)	
**Year**	2013	67 (4.1%)	37 (55%)	0.006***
	2014	341 (21%)	201 (59%)	
	2015	260 (16%)	131 (50%)	
	2016	357 (22%)	193 (54%)	
	2017	211 (13%)	142 (67%)	
	2018	410 (25%)	244 (60%)	
**Province**	Equateur	612 (37%)	350 (57%)	< 0.001***
	Kasai Oriental	309 (19%)	209 (68%)	
	Katanga	205 (12%)	149 (73%)	
	Kivu	366 (22%)	161 (44%)	
	Province Orientale	154 (9.4%)	79 (51%)	
**Place of residence**	Rural	1,462 (89%)	848 (58%)	0.100
	Urban	184 (11%)	100 (54%)	
	0–4	184 (11%)	133 (72%)	
**Years since last delivery**	5–9	33 (2.0%)	23 (70%)	< 0.001***
	≥ 10	45 (2.7%)	14 (31%)	
	Not applicable /Unknown	1,384 (84%)	778 (56%)	
**Fistula duration**	0–9 years	475 (29%)	319 (67%)	< 0.001***
	10–19 years	441 (27%)	291 (66%)	
	20 years or more	730 (44%)	338 (46%)	
**Number of abortions**	0	1,094 (66%)	701 (64%)	< 0.001***
	1–2	352 (21%)	178 (51%)	
	3 or more	83 (5.0%)	44 (53%)	
	Unknown	117 (7.1%)	25 (21%)	
**Number of surgeries before fistula repair**	0	818 (50%)	458 (56%)	0.004***
	1–2	704 (43%)	432 (61%)	
	3 or more	124 (7.5%)	58 (47%)	
**Parity**	Low (0–2 children)	800 (49%)	575 (72%)	< 0.001***
	Medium (3–4 children)	330 (20%)	205 (62%)	
	High (≥ 5 children)	516 (31%)	168 (33%)	

^1^Number of women (%), ^2^Pearson’s Chi-squared test

Statistical significance: ***p-value < 0.01, **p-value < 0.05, *p-value < 0.1

**Table 2:** Descriptive statistics and bivariate analysis of factors associated with the desire for children after fistula repair by parity level among all women aged 15–49

**Variables**	**Low parity (0–2 children)**		**Medium parity (3–4 children)**		**High parity (≥ 5 children)**	
	Overall	Number and % of women desiring to have children	Overall	Number and % of women desiring to have children	Overall	Number and % of women desiring to have children
	N^1^ (%)		N (%)		N (%)	
	800 (**100)**	**575 (71.9%)**	330 (**100)**	**205 (62.1%)**	516 (**100)**	**168 (32.6%)**
**Age**	***p*-value**^**2**^ < 0.001***		*p*-value < 0.001*** ^**X**^		*p*-value < 0.001*** ^**X**^	
15–19	119 (15%)	102 (86%)	8 (2.4%)	7 (88%)	13 (2.5%)	8 (62%)
20–24	200 (25%)	176 (88%)	50 (15%)	44 (88%)	54 (10%)	28 (52%)
25–29	133 17%)	112 (84%)	82 (25%)	62 (76%)	114 (22%)	58 (51%)
30–34	88 (11%)	64 (73%)	74 (22%)	45 (61%)	98 (19%)	44 (45%)
35–39	70 (8.8%)	56 (80%)	30 (9.1%)	23 (77%)	86 (17%)	16 (19%)
40–44	45 (5.6%)	27 (60%)	18 (5.5%)	10 (56%)	151 (29%)	14 (9.3%)
45–49	145 (18%)	38 (26%)	68 (21%)	14 (21%)		
**Marital status**	*p* -value = 0.002***		*p*-value = 0.049*		***p*-value = 0.2**	
Married	533 (67%)	387 (73%)	216 (65%)	134 (62%)	327 (63%)	116 (35%)
Separated/Divorced	134 (17%)	105 (78%)	60 (18%)	44 (73%)	108 (21%)	34 (31%)
Widow	59 (7.4%)	43 (73%)	28 (8.5%)	12 (43%)	49 (9.5%)	11 (22%)
Unknown	74 (9.3%)	40 (54%)	26 (7.9%)	15 (58%)	32 (6.2%)	7 (22%)
**Profession**	*p*-value < 0.001***		***p*-value = 0.11**		***p*-value = 0.8**	
Farmer	119 (15%)	102 (86%)	54 (16%)	37 (69%)	79 (15%)	23 (29%)
Housekeeper	494 (62%)	376 (76%)	234 (71%)	144 (62%)	395 (77%)	129 (33%)
Seller	30 (3.8%)	26 (87%)	9 2. (7%)	8 (89%)	11 (2.1%)	4 (36%)
Other/Unknown	157 (20%)	71 (45%)	33 (10%)	16 (48%)	31 (6.0%)	12 (39%)
**Religion**	*p*-value < 0.001***		***p*-value = 0.2**		*p*-value = 0.032**	
Catholic	200 (25%)	142 (71%)	95 (29%)	53 (56%)	181 (35%)	46 (25%)
Other	176 (22%)	84 (48%)	49 (15%)	28 (57%)	72 (14%)	24 (33%)
Protestant	424 (53%)	349 (82%)	186 (56%)	124 (67%)	263 (51%)	98 (37%)
**Highest diploma**	*p*-value < 0.001***		*p*-value = 0.008***		***p*-value = 0.13**	
Did not complete primary school	276 (35%)	196 (71%)	160 (48%)	89 (56%)	240 (47%)	83 (35%)
Completed primary school	307 (38%)	253 (82%)	124 (38%)	79 (64%)	211 (41%)	71 (34%)
Completed secondary school	217 (27%)	126 (58%)	46 (14%)	37 (80%)	65 (13%)	14 (22%)
**Year**	*p*-value < 0.001***		*p*-value = 0.021**		***p*-value = 0.7**	
2013	39 (4.9%)	24 (62%)	15 (4.5%)	9 (60%)	13 (2.5%)	4 (31%)
2014	153 (19%)	119 (78%)	70 (21%)	44 (63%)	118 (23%)	38 (32%)
2015	151 (19%)	83 (55%)	41 (12%)	26 (63%)	68 (13%)	22 (32%)
2016	158 (20%)	124 (78%)	75 (23%)	35 (47%)	124 (24%)	34 (27%)
2017	102 (13%)	85 (83%)	42 (13%)	33 (79%)	67 (13%)	24 (36%)
2018	197 (25%)	140 (71%)	87 (26%)	58 (67%)	126 (24%)	46 (37%)
**Province**	*p*-value < 0.001***		*p*-value = 0.048**		*p*-value = 0.011**	
Equateur	265 (33%)	212 (80%)	142 (43%)	79 (56%)	205 (40%)	59 (29%)
Kasai Oriental	152 (19%)	124 (82%)	63 (19%)	49 (78%)	94 (18%)	36 (38%)
Katanga	138 (17%)	108 (78%)	34 (10%)	22 (65%)	33 (6.4%)	19 (58%)
Kivu	181 (23%)	89 (49%)	56 (17%)	35 (63%)	129 ( 25%)	37 (29%)
Province Orientale	64 (8.0%)	42 (66%)	35 (11%)	20 (57%)	55 (11%)	17 (31%)
**Place of residence**	***p*-value = 0.2**		***p*-value = 0.4**		***p*-value = 0.2**	
Rural	710 (89%)	502 (79%)	297 (90%)	187 (63%)	455 (89%)	152 (34%)
Urban	90 (11%)	66 (73%)	33 (10%)	18 (55%)	61 (11%)	16 (26%)
**Years since last delivery**	*p*-value = 0.002***		*p*-value < 0.001***		*p*-value < 0.001***	
0–4	46 (5.8%)	41 (89%)	59 18%)	49 (83%)	79 (15%)	43 (54%)
5–9	9 (1.1%)	9 (100%)	10 (3.0%)	9 (90%)	14 (2.7%)	5 (36%)
≥ 10	12 (1.5%)	6 (50%)	13 (3.9%)	6 (46%)	20 (3.9%)	2 (10%)
Not applicable /Unknown	733 (92%)	41 (89%)	248 (75%)	141 (57%)	403 (78%)	118 (29%)
**Fistula duration**	*p*-value < 0.001***		***p*-value = 0.14**		***p*-value = 0.14**	
0–9 years	257 (32%)	213 (83%)	86 (26%)	61 (71%)	132 (26%)	45 (34%)
10–19 years	250 (31%)	199 (80%)	79 (24%)	48 (61%)	112 (22%)	44 (39%)
20 years or more	293 (37%)	163 (56%)	165 (50%)	96 (58%)	272 (53%)	79 (29%)
**Number of abortions**	*p*-value < 0.001***		*p*-value = 0.061*		***p*-value = 0.9**	
0	583 (73%)	462 (79%)	220 (67%)	141 (64%)	291 (56%)	98 (34%)
1–2	106 (13%)	84 (79%)	74 (22%)	41 (55%)	172 (33%)	53 (31%)
3 or more	15 (1.9%)	12 (80%)	23 (7.0%)	18 (78%)	45 (8.7%)	14 (31%)
Unknown	96 (12%)	17 (18%)	13 (3.9%)	5 (38%)	8 (1.6%)	3 (38%)
**Number of surgeries before fistula repair**	*p*-value < 0.001***		***p*-value = 0.4**		***p*-value = 0.2**	
0	406 (51%)	269 (66%)	160 (48%)	101 (63%)	252 (49%)	88 (35%)
1–2	343 (43%)	274 (80%)	135 (41%)	86 (64%)	226 (44%)	72 (32%)
3 or more	51 (6.4%)	32 (63%)	35 (11%)	18 (51%)	38 (7.4%)	8 (21%)

x: These chi-square test results should be interpreted with caution because some of the theoretical values are less than 5

Coverage: Women aged 15–49

^1^Number of women (%), ^2^Pearson’s Chi-squared test

Statistical significance: ***p-value < 0.01, **p-value < 0.05, *p-value < 0.1

Of the 1,646 women in the sample (Table 1), 948 (58%) wanted to have children after fistula repair. In bivariate analysis, all variables were significantly associated with the desire to have a child after fistula repair (*p* < 0.05). The proportion of women who wanted to have a child decreased with age (86% among women aged 15–19 years, 61% among those aged 30–34 years, and 18% among those aged 45–49 years). It also decreased with the number of years since the last delivery (from 72 to 31% for those who gave birth between 0 and 4 years and 10 years or more before fistula repair, respectively), the duration of the fistula (from 67 to 46% for those who had a fistula for less than 10 years and 20 years or more, respectively), and the number of abortions (from 64 to 53% for women who had a single abortion and three or more abortions, respectively). It was higher among women who have already had one to two operations (61%), divorced (61%), married (59%), sellers (76%), and farmers (64%). It was higher among women with primary education (63%) than among those with secondary education or no education (54%), among women whose fistula was repaired in 2017 (67%), in the provinces of Katanga (73%) and Kasai Oriental (68%), and among Protestant women (65%). The proportion of women who wished to have children decreased gradually according to the parity achieved at the time of fistula repair (72%, 62%, and 33% for women with low, medium, and high parity, respectively).

For each variable, the proportion of women who wished to have children decreased with increasing parity (Table 2). However, the association between the desire to have children and each variable was not always statistically significant, as we found for all parities combined. Among women with low parity, all variables, except place of residence (urban or rural), were significantly associated (*p* < 0.05) with the desire to have children. Among women with medium parity, the desire to have children varied significantly (*p* < 0.05) according to age, marital status, educational level, year of fistula repair, province of residence, and number of years since the last delivery. At last, among women with high parity, the desire to have children varied significantly (*p* < 0.05) according to age, religion, province, and number of years since their last delivery.

## Multivariate analysis of factors associated with the desire to have children after fistula repair for all women aged 15–49

Tables 3 and 4 show the results of the logistic model of factors associated with the desire to have children after fistula repair for all women aged 15–49 and by parity level.

**Table 3:** Logistic model of factors associated with fertility desires among fistula patients aged 15–49 (all parities combined)

**Characteristic**	**All women aged 20–34**		
	**aOR**^a^	**95% CI**^a^	***p*–value**
(Intercept)	6.51	2.34–18.6	< 0.001***
**Age** *(Reference: 15–19)*			
20–24	1.27	0.64–2.45	0.5
25–29	0.74	0.38–1.38	0.4
30–34	0.47	0.24–0.89	0.024**
35–39	0.54	0.26–1.08	0.089*
40–44	0.14	0.07–0.30	< 0.001***
45–49	0.06	0.03–0.12	< 0.001***
**Marital status** (*Reference: Married*)			
Separated	1.23	0.88–1.74	0.2
Widow	0.66	0.42–1.03	0.069*
Unknown	1.03	0.60–1.78	> 0.9
**Profession ***(Reference: Farmer)*			
Housekeeper	0.72	0.49–1.04	0.085*
Seller	1.04	0.46–2.48	> 0.9
Other/Unknown	0.64	0.36–1.14	0.13
**Religion** *(Reference: Catholic)*			
Other	1.32	0.87–2.01	0.2
Protestant	1.41	1.06–1.89	0.019**
**Highest diploma*** (Reference: Catholic)*			
Completed primary school	1.09	0.82–1.45	0.5
Completed secondary school	0.9	0.59–1.36	0.6
**Year** *(Reference: 2013)*			
2014	3.03	1.56–5.87	< 0.001***
2015	2.27	1.13–4.56	0.021**
2016	2.15	1.11–4.11	0.022**
2017	3.39	1.70–6.79	< 0.001***
2018	1.65	0.87–3.13	0.12
**Place of residence ***(Reference: Rural)*			
Urban	0.6	0.40–0.89	0.010**
**Years since last delivery** *(Reference: 0–4)*			
5–9	1.02	0.38–2.85	> 0.9
≥ 10	0.44	0.18–1.06	0.071*
Not applicable/Unknown	0.54	0.35–0.82	°0.004***
**Fistula duration** *(Reference: 0–9 years)*			
10–19 years	1.35	0.95–1.93	0.1
20 years or more	1.62	1.14–2.31	0.008***
**Number of abortions** *(Reference: 0)*			
1–2	1.15	0.84–1.58	0.4
3 or more	1.34	0.77–2.38	0.3
Unknown	0.44	0.21–0.90	°0.024**
**Number of surgeries before fistula repair** *(Reference: 0)*			
1–2	0.93	0.71–1.21	0.6
3 or more	0.45	0.28–0.72	< 0.001***
**Parity *(Reference: Low, 0–2 children)***			
Medium (3–4 children)	0.45	0.32–0.63	< 0.001***
High (≥ 5 children)	0.15	0.11–0.21	< 0.001***
Akaike information criterion (AIC)	1575.1		

^a^*aOR* Adjusted Odds Ratio, *CI* Confidence Interval. An odds ratio is significant at the 5% level if its confidence interval does not contain the value 1

Statistical significance: ***p-value < 0.01, **p-value < 0.05, *p-value < 0.1

°These results were not discussed because the “unknown” category does not provide information on the precise characteristics of the woman associated with the desire to have children

**Table 4:** Logistic model of factors associated with fertility desires among fistula patients aged 15–49 by parity level

**Characteristic**	**Low parity, 0–2 children (Model 1)**			**Medium parity, 3–4 children (Model 2)**			**High parity, ≥ 5 children (Model 3)**		
	**aOR**^a^	**95% CI**^a^	***p*–value**	**aOR**^a^	**95% CI**^a^	***p*–value**	**aOR**^a^	**95% CI**^a^	***p*–value**
(Intercept)	1.69	0.33–9.51	0.5	9.96	0.59–345	0.14	2.51	0.29–21.5	0.4
**Age** *(Reference: 15–19)*									
20–24	1.28	0.59–2.76	0.5	1.14	0.05–10.9	> 0.9	—	—	—
25–29	0.91	0.40–2.04	0.8	0.38	0.02–3.01	0.4	0.47	0.11–1.83	0.3
30–34	0.3	0.12–0.73	0.009***	0.21	0.01–1.64	0.2	0.49	0.13–1.75	0.3
35–39	0.64	0.22–1.90	0.4	0.41	0.02–3.85	0.5	0.34	0.08–1.22	0.11
40–44	0.13	0.05–0.37	< 0.001***	0.09	0.00–0.90	0.067*	0.1	0.02–0.39	0.001***
45–49	0.06	0.02–0.14	< 0.001***	0.03	0.00–0.23	0.004***	0.04	0.01–0.16	< 0.001***
**Marital status** (*Reference: Married*)									
Separated	1.14	0.64–2.11	0.7	1.93	0.86–4.52	0.12	1.02	0.58–1.77	> 0.9
Widow	0.84	0.41–1.80	0.6	0.52	0.19–1.37	0.2	0.45	0.19–1.00	0.058*
Unknown	3.49	1.18–12.9	°0.038**	1.09	0.36–3.38	0.9	0.51	0.17–1.36	0.2
**Profession ***(Reference: Farmer)*									
Housekeeper	0.73	0.37–1.39	0.4	0.56	0.24–1.25	0.2	0.88	0.47–1.68	0.7
Seller	1.27	0.33–5.90	0.7	1.98	0.22–46.0	0.6	0.69	0.15–2.88	0.6
Other/Unknown	0.59	0.24–1.46	0.3	0.28	0.08–1.00	0.053*	1.8	0.58–5.65	0.3
**Religion** *(Reference: Catholic)*									
Other	1.5	0.77–2.96	0.2	1.39	0.54–3.64	0.5	1.33	0.64–2.71	0.4
Protestant	1.66	1.03–2.69	0.037**	1.26	0.66–2.40	0.5	1.27	0.78–2.09	0.3
**Highest diploma*** (Reference: Catholic)*									
Completed primary school	1.95	1.21–3.17	0.006***	1.28	0.67–2.42	0.5	0.6	0.37–0.96	0.036**
Completed secondary school	1.61	0.85–3.13	0.2	2.25	0.79–7.00	0.14	0.28	0.12–0.62	0.002***
**Year** *(Reference: 2013)*									
2014	8.47	3.14–23.1	< 0.001***	1.72	0.42–6.95	0.4	2.06	0.50–9.55	0.3
2015	3.63	1.36–9.71	0.010**	1.06	0.23–4.73	> 0.9	2.55	0.56–12.8	0.2
2016	4.55	1.80–11.4	0.001***	0.86	0.21–3.41	0.8	1.5	0.37–6.95	0.6
2017	6.52	2.38–18.2	< 0.001***	2.46	0.52–11.8	0.3	2.17	0.52–10.2	0.3
2018	1.63	0.68–3.81	0.3	1.17	0.28–4.71	0.8	2.32	0.55–11.1	0.3
**Place of residence ***(Reference: Rural)*									
Urban	0.61	0.34–1.14	0.11	0.36	0.14–0.94	0.036**	0.64	0.30–1.31	0.2
**Years since last delivery** *(Reference: 0–4)*									
5–9	434,3	10–> 10^4^	> 0.9	3.65	0.41–88.0	0.3	0.61	0.15–2.31	0.5
≥ 10	0.35	0.06–1.87	0.2	0.99	0.18–5.30	> 0.9	0.26	0.03–1.37	0.15
Not applicable/Unknown	0.74	0.24–1.90	0.6	0.69	0.28–1.58	0.4	0.44	0.25–0.79	°0.006***
**Fistula duration** *(Reference: 0–9 years)*									
10–19 years	1.76	0.99–3.16	0.055*	0.82	0.35–1.93	0.7	1.77	0.94–3.35	0.076*
20 years or more	2.12	1.08–4.22	0.030**	1.46	0.67–3.22	0.3	1.46	0.84–2.57	0.2
**Number of abortions** *(Reference: 0)*									
1–2	1.28	0.69–2.46	0.4	1.09	0.53–2.30	0.8	1.18	0.72–1.93	0.5
3 or more	0.79	0.21–3.83	0.7	4.31	1.32–16.5	0.022**	0.8	0.34–1.78	0.6
Unknown	0.22	0.08–0.57	°0.002***	0.7	0.14–3.44	0.7	0.94	0.12–6.49	> 0.9
**Number of surgeries before fistula repair** *(Reference: 0)*									
1–2	0.9	0.58–1.39	0.6	0.81	0.43–1.51	0.5	0.96	0.60–1.52	0.9
3 or more	0.36	0.17–0.77	0.008***	0.43	0.17–1.10	0.079*	0.57	0.21–1.38	0.2
Akaike information criterion (AIC)	649.96			380.71			585.5		

^a^*aOR* Adjusted Odds ratio, *CI* Confidence Interval

Statistical significance: ***p-value < 0.01, **p-value < 0.05, *p-value < 0.1

°Not discussed for the same reason mentioned in Table 3

For all parities combined (Table 3), seven factors (parity, age, duration of fistula, number of surgeries, religion, year of fistula repair, and place of residence) were found to be significantly associated with fertility desire at the 5% level. The desire to have children gradually decreased with parity. It was significantly lower in women of medium parity (adjusted odds ratio, aOR = 0.45[0.32–0.63]) and in women of high parity (aOR = 0.15[0.11–0.21]) than in women of low parity. It decreases with age. It was significantly lower among women aged 40–44 years (aOR = 0.14, 95% CI = [0.07–0.30]) and those aged 45–49 years (aOR = 0.06[0.03–0.12]) than among those aged 15–19 years. The desire to have children increases with the duration of the fistula. It is significantly higher in women who have had a fistula for 20 years or more compared to those who have had a fistula for less than 10 years (aOR = 1.62[1.14–2.31]). The desire to have children was negatively associated with the number of surgeries performed prior to fistula repair. It is significantly lower in women who have had three or more surgeries than in those who have never undergone surgery (aOR = 0.45[0.28–0.72]). The desire for children was higher among Protestant women than among Catholic women (aOR = 1.41[1.06–1.89]). Globally, the desire for children tends to decrease over the years (aOR = 3.03[1.56–5.87]) in 2014 and aOR = 1.65[0.87–3.13] in 2018), but it was significantly higher in 2014 (aOR = 3.03[1.56–5.87]) and 2017 (aOR = 3.39[1.70–6.79]). It was lower in urban areas (aOR = 0.60[0.40–0.89]).

The results stratified by parity (Table 4, Models 1–3) show that the number of factors associated with the desire to have children decreases with parity: six, three and two factors were identified for women with low, medium and high parity, respectively. A comparison of the parity-specific models (Models 1–3, Table 4) and the model for all women (Table 3) reveals that most factors associated with the desire to have children vary considerably according to the parity level achieved by women. Parity-specific models (Table 4) fit the data better (lower AIC) than all-parity models (Table 3), supporting the premise that childbearing choices are made sequentially [25, 28]. Three trends emerge. First, certain associations observed in all women are present at all parity levels. This is the case for the negative association between age and the desire for children (Table 2, Models 1–4). Second, some factors (education and number of abortions) that did not seem to be associated with the desire to have children for all women are actually associated with the desire to have children for women of certain parity levels. Third, in most cases, certain factors (notably parity, religion, place of residence, and number of surgeries) that seemed to be associated with the desire to have children for all women are in fact associated with the desire to have children only for women of a certain parity level. Among women with low parity (Table 4, Model 1), the desire to have children after fistula repair is negatively associated with age (from aOR = 0.30[0.12–0.73] for women aged 30–34 to aOR = 0.06[0.02–0.14] for women aged 45–49), with a high number of surgeries before fistula repair (aOR = 0.36[0.17–0.77] for those with three or more surgeries), and with time period (from aOR = 8.47[3.14–23.1] in 2014 to aOR = 1.63[0.68–3.81] in 2018). It was high in Protestant women (aOR = 1.66[1.03–2.69]), women with less education (aOR = 1.95[1.21–3.17]), and women whose fistulas had lasted 20 years or more (aOR = 2.12[1.08–4.22]).

Among women with medium parity (Table 4, Model 2), the desire to have children was significantly lower in women aged 45–49 (aOR = 0.03[0.00–0.23]) and in urban areas (aOR = 0.36[0.14–0.94]). It was significantly higher (aOR = 4.31[1.32–16.5]) in women who had had more than two abortions compared with those who had never had an abortion.

Finally, among women with high parity (Table 4, Model 3), the desire to have children was considerably lower in those aged 40–44 and 45–49 (aOR = 0.10[0.02–0.39] and aOR = 0.04[0.01–0.16], respectively). It decreased with increasing level of education (from aOR = 0.60[0.37–0.96] to aOR = 0.28[0.12–0.62] in women with primary and secondary education, respectively).

### Multivariate analysis of factors associated with the desire to have children after fistula repair among women aged 20–34

For policy implications, we conducted regression analyses specifically focusing on women aged 20–34 (Table 5), and categorized them based on their parity level (Table 6).

**Table 5:** Logistic model of factors associated with fertility desires among fistula patients aged 20–34

**Characteristic**	**All women aged 20–34**		
	**(Model 1)**		
	**aOR**^a^	**95% CI**^a^	***p*–value**
(Intercept)	9.34	2.77–32.7	< 0.001***
**Age** *(Reference: 20–24)*			
25–29	0.52	0.31–0.86	0.012**
30–34	0.31	0.18–0.52	< 0.001***
**Marital status** (*Reference: Married*)			
Separated	1.08	0.67–1.75	0.8
Widow	0.52	0.28–0.97	0.037**
Unknown	0.84	0.42–1.76	0.6
**Profession ***(Reference: Farmer)*			
Housekeeper	0.90	0.50–1.55	0.7
Seller	0.65	0.22–2.03	0.4
Other/Unknown	0.52	0.24–1.11	0.090*
**Religion** *(Reference: Catholic)*			
Other	0.71	0.40–1.26	0.2
Protestant	1.20	0.78–1.82	0.4
**Highest diploma*** (Reference: Catholic)*			
Completed primary school	0.91	0.61–1.36	0.6
Completed secondary school	1.21	0.69–2.14	0.5
**Year** *(Reference: 2013)*			
2014	2.63	1.14–5.99	0.022**
2015	1.84	0.76–4.44	0.2
2016	2.09	0.92–4.73	0.076*
2017	3.17	1.30–7.79	0.011**
2018	1.43	0.64–3.13	0.4
**Place of residence ***(Reference: Rural)*			
Urban	0.64	0.38–1.11	0.11
**Years since last delivery** *(Reference: 0–4)*			
5–9	1.60	0.42–8.18	0.5
≥ 10	0.36	0.07–2.22	0.2
Not applicable/Unknown	0.57	0.34–0.96	°0.038**
**Fistula duration** *(Reference: 0–9 years)*			
10–19 years	1.37	0.88–2.15	0.2
20 years or more	1.58	0.98–2.56	0.060*
**Number of abortions** *(Reference: 0)*			
1–2	1.85	1.12–3.13	0.018**
3 or more	1.73	0.79–4.03	0.2
Unknown	1.16	0.42–3.64	0.8
**Number of surgeries before fistula repair** *(Reference: 0)*			
1–2	0.87	0.59–1.28	0.5
3 or more	0.46	0.24–0.86	0.015**
**Parity *(Reference: Low, 0–2 children)***			
Medium (3–4 children)	0.47	0.29–0.74	0.001***
High (≥ 5 children)	0.20	0.12–0.32	< 0.001***
Akaike information criterion (AIC)	832.72		

^a^*aOR* Adjusted Odds ratio, *CI* Confidence Interval

Statistical significance: ***p-value < 0.01, **p-value < 0.05, *p-value < 0.1

°Not discussed for the same reason mentioned in Table 3

**Table 6:** Logistic model of factors associated with fertility desires among fistula patients aged 20–34 by parity level

**Characteristic**	**Low parity, 0–2 children (Model 1)**			**Medium parity, 3–4 children (Model 2)**			**High parity, ≥ 5 children (Model 3)**		
	**aOR**^a^	**95% CI**^a^	***p*–value**	**aOR**^a^	**95% CI**^a^	***p*–value**	**aOR**^a^	**95% CI**^a^	***p*–value**
(Intercept)	14	1.13–395	0.061*	17	1.47–236	0.028	0.81	0.05–12.0	0.9
**Age** *(Reference: 20–24)*									
25–29	0.56	0.26–1.20	0.13	0.32	0.09–0.96	0.054*	0.66	0.15–2.63	0.6
30–34	0.19	0.08–0.45	< 0.001***	0.17	0.05–0.51	0.003***	0.63	0.16–2.26	0.5
**Marital status** (*Reference: Married*)									
Separated	1.07	0.46–2.65	0.9	2.06	0.70–6.85	0.2	0.84	0.37–1.91	0.7
Widow	1.44	0.46–5.63	0.6	0.16	0.03–0.63	0.012**	0.3	0.09–0.95	0.046**
Unknown	1.14	0.34–5.26	0.8	0.82	0.23–3.08	0.8	0.65	0.15–2.92	0.6
**Profession ***(Reference: Farmer)*									
Housekeeper	0.52	0.16–1.38	0.2	0.48	0.12–1.57	0.2	2.24	0.71–7.58	0.2
Seller	0.31	0.05–2.57	0.2	1.57	0.10–49.9	0.8	0.41	0.02–4.39	0.5
Other/Unknown	0.27	0.07–0.99	0.053*	0.4	0.07–2.02	0.3	2.67	0.46–16.5	0.3
**Religion** *(Reference: Catholic)*									
Other	0.49	0.19–1.23	0.13	1.38	0.39–5.21	0.6	0.88	0.28–2.72	0.8
Protestant	1.25	0.57–2.69	0.6	1.37	0.58–3.20	0.5	1.28	0.58–2.84	0.5
**Highest diploma*** (Reference: Catholic)*									
Completed primary school	1.93	0.95–3.98	0.071*	0.79	0.33–1.86	0.6	0.45	0.21–0.92	0.032**
Completed secondary school	2.7	1.10–7.14	0.036**	1.46	0.42–5.58	0.6	0.4	0.12–1.24	0.11
**Year** *(Reference: 2013)*									
2014	5.23	1.39–19.9	0.014**	2.38	0.42–13.2	0.3	3.09	0.59–17.8	0.2
2015	4.15	1.07–16.5	0.039**	0.89	0.14–5.66	> 0.9	2.19	0.34–15.1	0.4
2016	5.27	1.42–19.9	0.013**	1.03	0.19–5.48	> 0.9	1.76	0.35–9.48	0.5
2017	8.3	1.94–40.5	0.006***	4.03	0.55–34.2	0.2	2.06	0.38–11.9	0.4
2018	1.86	0.57–5.83	0.3	1	0.17–5.45	> 0.9	2.13	0.39–12.6	0.4
**Place of residence ***(Reference: Rural)*									
Urban	0.66	0.28–1.69	0.4	0.36	0.11–1.21	0.093*	0.49	0.16–1.43	0.2
**Years since last delivery** *(Reference: 0–4)*									
5–9	227,24	0.00– > 10^4^	> 0.9	5.51	0.42–229	0.3	0.59	0.06–6.17	0.6
≥ 10	0.03	0.00–0.62	0.030**	2,116	0.00– > 10^4^	> 0.9	0.42	0.01–14.8	0.6
Not applicable/Unknown	0.16	0.01–0.85	0.085*	0.65	0.25–1.63	0.4	0.77	0.33–1.76	0.5
**Fistula duration** *(Reference: 0–9 years)*									
10–19 years	1.71	0.82–3.68	0.2	1.24	0.45–3.49	0.7	1.66	0.71–4.00	0.2
20 years or more	2.43	0.92–6.88	0.082*	1.95	0.73–5.34	0.2	1.49	0.62–3.65	0.4
**Number of abortions** *(Reference: 0)*									
1–2	3.56	1.19–13.9	0.039**	0.9	0.31–2.74	0.8	1.32	0.56–3.17	0.5
3 or more	1.34	0.24–11.3	0.8	6.5	1.06–127	0.092*	0.83	0.24–2.86	0.8
Unknown	0.46	0.11–2.13	0.3	2.73	0.29–63.1	0.4	1.76	0.17–43.1	0.7
**Number of surgeries before fistula repair** *(Reference: 0)*									
1–2	1.28	0.65–2.55	0.5	0.49	0.21–1.16	0.11	0.71	0.34–1.45	0.4
3 or more	0.57	0.21–1.68	0.3	0.37	0.11–1.26	0.11	0.32	0.06–1.39	0.15
Akaike information criterion(AIC)	338.44			244.86			282.9		

^a^*aOR* Adjusted Odds ratio, *CI* Confidence Interval

Statistical significance: ***p–value < 0.01, **p–value < 0.05, *p–value < 0.1

Considering all women aged 20–34 (Table 5), six factors were significantly associated with the desire to have children after fistula repair. The desire to have children decreases with parity (aOR = 0.47[0.29–0.74] and aOR = 0.20[0.12–0.32] in women with medium and high parity, respectively). It is significantly lower at older ages (aOR = 0.52[0.31–0.86] and aOR = 0.31[0.18–0.52] in women aged 25–29 and 30–34, respectively), in widows (aOR = 0.52[0.28–0.97]), and in women who have undergone three or more surgeries (aOR = 0.46[0.24–0.86]). It tended to decrease over time (aOR = 2.63[1.14–5.99] in 2014 to aOR = 1.43[0.64–3.13] in 2018), but was particularly high in 2014 and 2017 (aOR = 3.17[1.30–7.79]). It was significantly higher among women who have had one to two abortions (aOR = 1.85[1.12–3.13]).

A comparison of these results with those stratified by parity shows that the association between fertility desire and its factors among women aged 20–34 is also parity-specific (Table 6). First, two factors (educational attainment and years since last childbirth) that do not appear to be associated with fertility desire for all parities combined are actually associated with fertility desire at certain parity levels. Second, one factor (number of surgeries prior to fistula repair) was associated with fertility desire only when all parity levels were combined. At last, four factors (age, marital status, time, and number of abortions) that appear to be associated with fertility desire for all women are actually associated with women’s fertility desire only at a specific parity level.

Among women with low parity (Table 6, Model 1), the desire to have children was lower among women aged 30–34 years (aOR = 0.19[0.08–0.45]). It was higher among women who had completed secondary education (aOR = 2.70[1.10–7.14]), women who underwent surgery in 2014 (aOR = 5.23[1.39–19.9]) and 2017 (aOR = 8.30[1.94–40.5]), and among women who had one to two abortions (aOR = 3.56[1.19–13.9]). Among women of medium parity (Table 6, Model 2), the desire to have children is significantly lower among women aged 30–34 (aOR = 0.17[0.05–0.51]) and among widows (aOR = 0.16[0.03–0.63]). Finally, among women with high parity (Table 6, Model 3), the desire to have children was significantly lower among widows (aOR = 0.30[0.09–0.95]) and women who had completed primary school (aOR = 0.17[0.05–0.51]).

## Discussion

The proportion of women aged 15–49 years who wished to have a child after fistula repair was 57.6%. This is lower than the 64.7% reported by Benfield et al. [16], using a sample of 61 women. The small sample size may explain this difference in results. It is lower than that of all Congolese women aged 15–49 years for the same period (76.6% in 2013/2014) [4]. The despair of not being able to heal the fistula may be the reason for the low desire for children among women with fistula. In the most fertile age group (20–34 years), 73.8% of women wanted children after fistula repair, indicating that this group needs special attention.

Other studies have found that fertility desire is negatively associated with increasing parity [15, 23]. We also found the same negative association between fertility desire and actual parity among both women aged 15–49 and those aged 20–34. This finding can be explained by the fact that as parity increases, a woman gets closer to her ideal number of children and the desire for an additional child decreases. Therefore, we stratified all regression analyses by parity level.

Six factors (number of surgeries, duration of fistula, age, year of repair, religion, and place of residence) in women aged 15–49 and six factors in women aged 20–34 (number of surgeries, abortions, age, year of repair, marital status, level of education) were associated with parity-specific desires for children. Parity-specific findings showed that a woman’s parity level affects the relationship between desire for children and its correlates. Thus, the relationship between the desire to have children and religion, level of education, year of fistula repair, duration of fistula, number of surgeries (Table 4), and number of abortions (Table 6) was stronger among women with low parity. In addition, most of the factors were associated with fertility desire in women with low parity (Tables 4 and 6) suggesting that this group is a suitable target for possible interventions.

The desire to have children after fistula repair was negatively associated with the number of surgical operations performed among women aged 15–49 years and 20–34 years. This finding may be explained by the fear of having to undergo another operation during the next childbirth, as some studies have shown [29]. This relationship was not observed across parity levels in women aged 20–34 years. However, it was found among all women aged 15–49 years with low parity. This finding highlights the constraining effect of the number of operations a woman already had on the demand for children. Even women who have no or fewer children do not want more children because of previous surgeries. According to our results, the desire to have children is higher among women who have had fistulas for a long time. It was high among all women of reproductive age with fistula for 20 years or more, especially among those with low parity. This finding underscores the need to help women with long-standing fistulas achieve their ideal family sizes. We found a high desire for children among high parity women aged 15–49 who had three or more abortions, and among low parity women aged 20–34 who had one to two abortions. The abortions these women have had, most likely as a result of fistula [9], have reduced their fertility and pushed them further away from their ideal family size, increasing their desire for children.

Fertility desire was negatively associated with age (in women aged 15–49 and 20–35). This relationship was observed across all the parity levels. This finding suggests that women are aware of the decline in their biological capacity to reproduce as they age [30]. In our literature review, we found no multivariate or explanatory studies on the factors associated with the desire to have children among women with fistula in the DRC. Studies on other categories of patients suffering from long-term illnesses or infections such as HIV [31] have shown that the desire to have children decreases with age.

Among all women aged 15–49 with medium or high parity, the desire to have children does not vary by religion, as they would already be closer to their ideal family size. For women with low parity, we found an association between religious affiliation and the desire to have children, that is, a higher desire to have children among Protestant women than among Catholic women. This finding is consistent with that of Mosuse and Gadeyne [32], who found a high desire for a large number of children among Protestants, other Christians, and Muslims in the DRC. This finding highlights the importance of procreation and reproduction in messages taught by the Congolese Protestant Church [33]. In fact, the teachings within the Protestant Church are not standardized, as each leader or pastor preaches the topic of their choice and approaches it according to their understanding. For example, some preachers preach that family planning is a sin and that God punishes those who practice it [33]. Although the same point of view is present among Catholics, this type of message is taught much less often because the theme of teaching every Sunday is the same in all churches.

The desire to have children tended to decrease over time. It was lower among women whose fistulas were repaired in 2018 than among those whose fistulas had been repaired in previous years, particularly in 2014 and 2017. This relationship was found for both women aged 20–34 years and women aged 15–49 years but only for women with low parity. This finding was not limited to women with fistula. In fact, a decline in the proportion of women in the DRC who wanted to have a child was also reported in the 2007 (80.3%) and 2013/2014 (76.6%) Demographic and Health Surveys [4, 34].

Rural women aged 15–49 years, especially those with medium parity, had a higher desire to have children than urban women. This result is not very different from that observed for all Congolese women in 2013/2014, which shows that a large proportion of women want more children in rural settings (79.4% compared to 70.7% in urban settings) [4]. Several hypotheses have been proposed to explain this result. First, having a large number of children is advantageous because they contribute to the family’s agricultural activity [35]. Second, having many children is seen as insurance for old age [36].

We found a lower desire for children among widows of medium or high parity aged 20–34 years compared to married women. Widows may be desperate to find husbands, given their condition, or they may not want children because they lack the means to care for them. Ultimately, they may find contentment with their limited number of children, regardless of whether they have achieved their desired number of children. Finally, fertility desire was higher among women of low parity who completed primary or secondary school compared to those who did not complete primary school. This finding means that education does not play its role in reducing the demand for children, as found in other studies [37], among women with fewer or no children. However, among women with high parity, the desire for children was lower among those who completed primary or secondary school. This finding is in line with other studies conducted on all women of reproductive age in Sub-Saharan Africa [37]. One possible explanation is that highly educated women sometimes find it difficult to reconcile having several children with life goals such as certain skilled occupations or managerial positions.

## Strengths and limitations

The first strength of this study is its larger sample size compared to other studies in the DRC on fertility desires of women with fistula. Its second strength is its multivariate descriptive approach compared to other studies on the DRC, which are univariate descriptive.

The first limitation is the lack of information that was not completed or to which the patients did not respond, such as the number of abortions. The effect of this variable must be interpreted with caution, since the missing category had a statistically significant association with fertility desire. For other variables, the unknown category was not significantly associated with women’s fertility desire. Nevertheless, missing information does not disrupt the sample structure and does not affect our overall findings, as no data were deleted. Another limitation of this study is its cross-sectional nature, which makes it impossible to establish cause-and-effect relationships between fertility desires and the factors identified. In addition, fertility desires and intentions may change over time for the same individual [38], underscoring the importance of longitudinal studies that follow women to assess whether their fertility desires or intentions have been fulfilled.

## Conclusion

Our findings indicated that the correlates of fertility desire after fistula repair among women aged 15–49 or 20–34 years were parity-specific. Desire for children was negatively associated with age across all parity levels. Among women with low parity, it was significantly and negatively associated with a high number of surgeries, number of abortions, and fistula duration. It tended to decrease with time but was particularly high in 2014 and 2017. It was high among the Protestant women. Among women with medium parity, it was significantly lower in urban areas and among widows but higher among in who had more than two abortions. Among high-parity women, it was negatively associated with education level.

From a policy perspective, these factors can be divided into two categories. On the one hand, there are factors that can help identify subpopulations of women who have a high desire to have children after fistula repair and therefore need counseling, such as women with low or medium parity under 25 years of age, married women with medium or high parity, low-parity women who had surgery in 2014 and 2017, low-parity women who had been childless for at least 10 years, and low-parity women whose fistula has lasted 20 years or more. On the other hand, there are factors that can be taken into consideration to help fistula patients achieve their ideal family size without compromising their health, including the number of abortions and the number of surgeries. Possible interventions include (1) training medical staff to reduce the number of unsuccessful surgeries performed on women with fistula; (2) providing the human, material, and financial resources needed to eliminate fistula in the DRC; (3) providing prenatal counseling and family planning services throughout the DRC and making these services affordable; and (4) educating women about the importance of attending these services and raising their awareness of the proper attitudes to adopt during pregnancy.

### Supplementary Information

Supplementary Material 1: Supplementary Table 1. Colinearity diagnosis. Supplementary Table 2. Descriptive statistics and bivariate analysis of factors associated with the desire for children after fistula repair (women aged 20–34). Supplementary Table 3. Logistic model (including interaction between year of fistula repair and age) of factors associated with fistula desires for women aged 15–49. Supplementary Table 4. Logistic model (including interaction between year of fistula repair and age) of factors associated with fistula desires for women aged 20–35.

## Data Availability

The data used in this study are available upon request to Panzi hospital.

## Abbreviations

DRC: Democratic Republic of Congo
aOR: Adjusted odds-ratio
CI: Confidence interval
