# Supplementary material for: Correlates of fertility desires in women with urogenital fistula in the Democratic Republic of Congo: a cross-sectional study of 1,646 women
Source: Reprod Health. 2024 Jul 3;21:99. doi: 10.1186/s12978-024-01823-z (PMC11221031; doi:10.1186/s12978-024-01823-z)
Supplement: Supplementary file 1 — Supplementary Material 1: Supplementary Table 1. Colinearity diagnosis. Supplementary Table 2. Descriptive statistics and bivariate analysis of factors associated with the desire for children after fistula repair (women aged 20–34). Supplementary Table 3. Logistic model (including interaction between year of fistula repair and age) of factors associated with fistula desires for women aged 15–49. Supplementary Table 4. Logistic model (including interaction between year of fistula repair and age) of factors associated with fistula desires for women aged 20–35. [file 12978_2024_1823_MOESM1_ESM.docx]

**Appendices**

*Supplementary Table 1. Colinearity diagnosis*

|  | Model with the collinear variable, "Province" | | | Model without the collinear variable, "Province" | | |
| --- | --- | --- | --- | --- | --- | --- |
| **Variables** | **GVIF** | **Df** | **GVIF^(1/(2*Df))** | **GVIF** | **Df** | **GVIF^(1/(2*Df))** |
| Age | 2.229 | 6 | 1.069 | 2.182 | 6 | 1.0672 |
| Marital status | 1.117 | 3 | 1.019 | 1.091 | 3 | 1.0147 |
| Profession | 1.466 | 3 | 1.066 | 1.374 | 3 | 1.0543 |
| Religion | 1.184 | 2 | 1.043 | 1.131 | 2 | 1.0312 |
| Highest diploma | 1.358 | 2 | 1.080 | 1.283 | 2 | 1.0642 |
| Year | 2.951 | 5 | 1.114 | 1.447 | 5 | 1.0376 |
| Place of residence | 1.097 | 1 | 1.047 | 1.086 | 1 | 1.0422 |
| Province | 3.174 | 4 | 1.155 |  |  |  |
| Years since last delivery | 1.245 | 3 | 1.037 | 1.207 | 3 | 1.0319 |
| Fist.duration | 1.603 | 2 | 1.125 | 1.526 | 2 | 1.1114 |
| Number of abortions | 1.269 | 3 | 1.041 | 1.262 | 3 | 1.0395 |
| `Number of operations | 1.107 | 2 | 1.026 | 1.087 | 2 | 1.0211 |
| Parity | 1.473 | 2 | 1.102 | 1.424 | 2 | 1.0924 |
| Note: ^(1/(2×𝐷𝑓))^ (the square root of the VIF/GVIF value as DF = 1) is the proportional change in the standard error and confidence interval of its coefficients due to the degree of collinearity. The (1/(2×𝐷𝑓)) value of the categorical variable is a similar measure of the reduction in the precision of the estimation of the coefficients due to collinearity. | | | | | | |

*Supplementary Table 2. Descriptive statistics and bivariate analysis of factors associated with the desire for children after fistula repair (women aged 20–34)*

| **Characteristic** | **Overall,**  **N = 808*^1^*** | **Women not wanting more children, N = 211*^1^*** | **Women wanting more children, N = 597*^1^*** | **p-value*^2^*** |
| --- | --- | --- | --- | --- |
| **Age** |  |  |  | **<0.001***** |
| 20–24 | 263 (33%) | 35 (17%) | 228 (38%) |  |
| 25–29 | 269 (33%) | 67 (32%) | 202 (34%) |  |
| 30–34 | 276 (34%) | 109 (52%) | 167 (28%) |  |
| **Marital_status** |  |  |  | **0.036*** |
| Married | 532 (66%) | 132 (63%) | 400 (67%) |  |
| Separated | 155 (19%) | 36 (17%) | 119 (20%) |  |
| Widow | 64 (7.9%) | 26 (12%) | 38 (6.4%) |  |
| Undefined | 57 (7.1%) | 17 (8.1%) | 40 (6.7%) |  |
| **Profession** |  |  |  | 0.14 |
| Farmer | 118 (15%) | 23 (11%) | 95 (16%) |  |
| Housekeeper | 567 (70%) | 149 (71%) | 418 (70%) |  |
| Other/Unknown | 95 (12%) | 32 (15%) | 63 (11%) |  |
| Seller | 28 (3.5%) | 7 (3.3%) | 21 (3.5%) |  |
| **Religion** |  |  |  | **0.002***** |
| Catholic | 205 (25%) | 58 (27%) | 147 (25%) |  |
| Other | 126 (16%) | 47 (22%) | 79 (13%) |  |
| Protestant | 477 (59%) | 106 (50%) | 371 (62%) |  |
| **Highest diploma** |  |  |  | 0.8 |
| No education | 303 (38%) | 80 (38%) | 223 (37%) |  |
| Primary | 334 (41%) | 90 (43%) | 244 (41%) |  |
| Secondary | 171 (21%) | 41 (19%) | 130 (22%) |  |
| **Year** |  |  |  | 0.065* |
| 2013 | 43 (5.3%) | 17 (8.1%) | 26 (4.4%) |  |
| 2014 | 169 (21%) | 40 (19%) | 129 (22%) |  |
| 2015 | 113 (14%) | 35 (17%) | 78 (13%) |  |
| 2016 | 163 (20%) | 42 (20%) | 121 (20%) |  |
| 2017 | 109 (13%) | 19 (9.0%) | 90 (15%) |  |
| 2018 | 211 (26%) | 58 (27%) | 153 (26%) |  |
| **Province** |  |  |  | **0.019**** |
| Equateur | 273 (34%) | 61 (29%) | 212 (36%) |  |
| Kasai Orient. | 179 (22%) | 40 (19%) | 139 (23%) |  |
| Katanga | 122 (15%) | 30 (14%) | 92 (15%) |  |
| Kivu | 167 (21%) | 59 (28%) | 108 (18%) |  |
| Province Orient. | 67 (8.3%) | 21 (10.0%) | 46 (7.7%) |  |
| **Place of residence** |  |  |  | 0.11 |
| Rural | 711 (88%) | 180 (85%) | 531 (89%) |  |
| Urban | 97 (12%) | 31 (15%) | 66 (11%) |  |
| **Years since last delivery** |  |  |  | 0.3 |
| 0–4 | 130 (16%) | 28 (13%) | 102 (17%) |  |
| 5-9 | 19 (2.4%) | 3 (1.4%) | 16 (2.7%) |  |
| ≥10 | 8 (1.0%) | 3 (1.4%) | 5 (0.8%) |  |
| Not applicable/Unknown | 651 (81%) | 177 (84%) | 474 (79%) |  |
| **Fistula duration** |  |  |  | >0.9 |
| 0-9 | 314 (39%) | 80 (38%) | 234 (39%) |  |
| 10-19 | 264 (33%) | 70 (33%) | 194 (32%) |  |
| 20 or more | 230 (28%) | 61 (29%) | 169 (28%) |  |
| **Number of abortions** |  |  |  | 0.079* |
| 0 | 604 (75%) | 157 (74%) | 447 (75%) |  |
| 1–2 | 131 (16%) | 28 (13%) | 103 (17%) |  |
| 3 or more | 38 (4.7%) | 11 (5.2%) | 27 (4.5%) |  |
| Unknown | 35 (4.3%) | 15 (7.1%) | 20 (3.4%) |  |
| **Number of operations** |  |  |  | **0.048**** |
| 0 | 381 (47%) | 94 (45%) | 287 (48%) |  |
| 1–2 | 363 (45%) | 92 (44%) | 271 (45%) |  |
| 3 or more | 64 (7.9%) | 25 (12%) | 39 (6.5%) |  |
| **Parity** |  |  |  | **<0.001***** |
| Low (0–2) | 421 (52%) | 69 (33%) | 352 (59%) |  |
| Medium (3–4) | 206 (25%) | 55 (26%) | 151 (25%) |  |
| High (≥5) | 181 (22%) | 87 (41%) | 94 (16%) |  |
| *^1^* n (%), ^2^Pearson’s Chi-squared test  Statistical significance : ***p-value<0.01, **p-value<0.05, *p-value<10% | | | | |

*Supplementary Table 3. Logistic model (including interaction between year of fistula repair and age) of factors associated with fistula desires for women aged 15-49*

| **Characteristic** | **log(OR)***^1^* | **95% CI***^1^* | **p-value** |
| --- | --- | --- | --- |
| (Intercept) | 1.5 | -0.26, 3.3 | 0.10 |
| Age |  |  |  |
| 15-19 | — | — |  |
| 20-24 | 0.45 | -1.8, 2.7 | 0.7 |
| 25-29 | 0.32 | -1.7, 2.2 | 0.7 |
| 30-34 | 0.12 | -1.9, 2.1 | >0.9 |
| 35-39 | 16 | 573, 518 | >0.9 |
| 40-44 | -17 | -1525 | >0.9 |
| 45-49 | -2.1 | -5.4, 0.40 | 0.13 |
| Year |  |  |  |
| 2013 | — | — |  |
| 2014 | 16 | 348, 350 | >0.9 |
| 2015 | 1.5 | -0.70, 3.8 | 0.2 |
| 2016 | 2.3 | -0.08, 5.6 | 0.078 |
| 2017 | 2.6 | 0.19, 5.8 | 0.050 |
| 2018 | 0.39 | -1.5, 2.2 | 0.7 |
| Marital_status |  |  |  |
| 1 Married | — | — |  |
| 3 Separated | 0.11 | -0.23, 0.45 | 0.5 |
| 4 Widow | -0.44 | -0.90, 0.01 | 0.055 |
| 5 Undefined | -0.14 | -0.68, 0.41 | 0.6 |
| Profession |  |  |  |
| Farmer | — | — |  |
| Housekeeper | -0.36 | -0.74, 0.01 | 0.056 |
| Other/Unknown | -0.35 | -0.92, 0.22 | 0.2 |
| Seller | -0.09 | -0.92, 0.80 | 0.8 |
| Religion |  |  |  |
| Catholic | — | — |  |
| Muslim | 1.1 | -0.58, 2.8 | 0.2 |
| Other | 0.16 | -0.26, 0.59 | 0.4 |
| Protestant | 0.35 | 0.06, 0.64 | 0.017 |
| Highest diploma |  |  |  |
| No education | — | — |  |
| Primary | 0.00 | -0.28, 0.28 | >0.9 |
| Secondary | -0.19 | -0.62, 0.23 | 0.4 |
| Place of residence |  |  |  |
| Rural | — | — |  |
| Urban | -0.48 | -0.87, -0.09 | 0.016 |
| Parity3 |  |  |  |
| 0-1 | — | — |  |
| 2 or more | -1.3 | -1.7, -1.0 | <0.001 |
| Years since last delivery |  |  |  |
| 0-4 | — | — |  |
| Ten or more | -0.26 | -1.1, 0.58 | 0.5 |
| 5-9 | 0.22 | -0.69, 1.2 | 0.6 |
| Not applicable/Unknown | -0.45 | -0.87, -0.04 | 0.031 |
| Fist.duration |  |  |  |
| 0-9 | — | — |  |
| 10-19 | 0.37 | 0.01, 0.74 | 0.045 |
| 20 or more | 0.57 | 0.22, 0.93 | 0.002 |
| Number of abortions |  |  |  |
| 0 | — | — |  |
| 1-2 | 0.00 | -0.31, 0.30 | >0.9 |
| 3 or more | -0.06 | -0.62, 0.50 | 0.8 |
| Unknown | -0.92 | -1.7, -0.18 | 0.015 |
| Number of operations |  |  |  |
| 0 | — | — |  |
| 1-2 | -0.07 | -0.34, 0.20 | 0.6 |
| 3 or more | -0.68 | -1.2, -0.20 | 0.006 |
| Age * Year |  |  |  |
| 20-24 * 2014 | -14 | -609 | >0.9 |
| 25-29 * 2014 | -16 | -635 | >0.9 |
| 30-34 * 2014 | -15 | -708 | >0.9 |
| 35-39 * 2014 | -32 | -1470 | >0.9 |
| 40-44 * 2014 | -0.14 | -7.3, 7.4 | >0.9 |
| 45-49 * 2014 | -15 | -729 | >0.9 |
| 20-24 * 2015 | -0.76 | -3.7, 2.0 | 0.6 |
| 25-29 * 2015 | -0.49 | -3.2, 2.1 | 0.7 |
| 30-34 * 2015 | -1.2 | -3.9, 1.3 | 0.3 |
| 35-39 * 2015 | -17 | -1104 | >0.9 |
| 40-44 * 2015 | 15 | 843, 833 | >0.9 |
| 45-49 * 2015 | -2.0 | -5.2, 1.7 | 0.2 |
| 20-24 * 2016 | -0.49 | -4.1, 2.5 | 0.8 |
| 25-29 * 2016 | -1.3 | -4.7, 1.4 | 0.4 |
| 30-34 * 2016 | -2.3 | -5.7, 0.36 | 0.11 |
| 35-39 * 2016 | -18 | -1295 | >0.9 |
| 40-44 * 2016 | 13 | 843, 657 | >0.9 |
| 45-49 * 2016 | -2.1 | -5.8, 1.6 | 0.2 |
| 20-24 * 2017 | 0.45 | -3.4, 4.3 | 0.8 |
| 25-29 * 2017 | -1.5 | -4.9, 1.3 | 0.3 |
| 30-34 * 2017 | -2.2 | -5.6, 0.60 | 0.15 |
| 35-39 * 2017 | -17 | -1182 | >0.9 |
| 40-44 * 2017 | 12 | 862, 867 | >0.9 |
| 45-49 * 2017 | -1.4 | -5.2, 2.3 | 0.4 |
| 20-24 * 2018 | 0.64 | -1.8, 3.1 | 0.6 |
| 25-29 * 2018 | 0.30 | -1.9, 2.5 | 0.8 |
| 30-34 * 2018 | -0.52 | -2.7, 1.7 | 0.6 |
| 35-39 * 2018 | -16 | -1080 | >0.9 |
| 40-44 * 2018 | 15 | 792, 745 | >0.9 |
| 45-49 * 2018 | 0.10 | -2.6, 3.5 | >0.9 |
| *^1^* OR = Odds Ratio, CI = Confidence Interval | | | |

*Supplementary Table 4. Logistic model (including interaction between year of fistula repair and age) of factors associated with fistula desires for women aged 20-35*

| **Characteristic** | **log(aOR)***^1^* | **95% CI***^1^* | **p-value** |
| --- | --- | --- | --- |
| (Intercept) | 2.0 | 0.26, 3.9 | 0.029 |
| Age |  |  |  |
| 20-24 | — | — |  |
| 25-29 | -0.29 | -2.1, 1.4 | 0.7 |
| 30-34 | -0.78 | -2.7, 0.97 | 0.4 |
| Year |  |  |  |
| 2013 | — | — |  |
| 2014 | 1.4 | -0.37, 3.1 | 0.10 |
| 2015 | 0.52 | -1.3, 2.2 | 0.6 |
| 2016 | 1.5 | -0.34, 3.3 | 0.10 |
| 2017 | 2.7 | 0.47, 5.9 | 0.030 |
| 2018 | 0.88 | -0.85, 2.4 | 0.3 |
| Marital_status |  |  |  |
| 1 Married | — | — |  |
| 3 Separated | -0.09 | -0.56, 0.39 | 0.7 |
| 4 Widow | -0.76 | -1.4, -0.14 | 0.015 |
| 5 Undefined | -0.23 | -0.92, 0.50 | 0.5 |
| Profession |  |  |  |
| Farmer | — | — |  |
| Housekeeper | -0.21 | -0.77, 0.33 | 0.5 |
| Other/Unknown | -0.56 | -1.3, 0.19 | 0.14 |
| Seller | -0.53 | -1.6, 0.59 | 0.3 |
| Religion |  |  |  |
| Catholic | — | — |  |
| Muslim | 0.48 | -2.3, 3.8 | 0.7 |
| Other | -0.36 | -0.94, 0.22 | 0.2 |
| Protestant | 0.16 | -0.27, 0.57 | 0.5 |
| Highest diploma |  |  |  |
| No education | — | — |  |
| Primary | -0.18 | -0.58, 0.22 | 0.4 |
| Secondary | 0.20 | -0.35, 0.77 | 0.5 |
| Place of residence |  |  |  |
| Rural | — | — |  |
| Urban | -0.38 | -0.91, 0.16 | 0.2 |
| Parity3 |  |  |  |
| 0-1 | — | — |  |
| 2 or more | -0.90 | -1.4, -0.46 | <0.001 |
| Years since last delivery |  |  |  |
| 0-4 | — | — |  |
| Ten or more | -0.92 | -2.5, 0.85 | 0.3 |
| 5-9 | 0.76 | -0.57, 2.4 | 0.3 |
| Not applicable/Unknown | -0.38 | -0.91, 0.13 | 0.2 |
| Fist.duration |  |  |  |
| 0-9 | — | — |  |
| 10-19 | 0.34 | -0.11, 0.79 | 0.15 |
| 20 or more | 0.43 | -0.04, 0.91 | 0.074 |
| Number of abortions |  |  |  |
| 0 | — | — |  |
| 1-2 | 0.54 | 0.05, 1.1 | 0.037 |
| 3 or more | 0.21 | -0.56, 1.0 | 0.6 |
| Unknown | 0.02 | -0.99, 1.2 | >0.9 |
| Number of operations |  |  |  |
| 0 | — | — |  |
| 1-2 | -0.17 | -0.56, 0.22 | 0.4 |
| 3 or more | -0.74 | -1.4, -0.09 | 0.024 |
| Age * Year |  |  |  |
| 25-29 * 2014 | -1.3 | -3.4, 0.81 | 0.2 |
| 30-34 * 2014 | -0.21 | -2.3, 1.9 | 0.8 |
| 25-29 * 2015 | 0.35 | -1.8, 2.6 | 0.7 |
| 30-34 * 2015 | -0.05 | -2.2, 2.2 | >0.9 |
| 25-29 * 2016 | -0.70 | -2.9, 1.5 | 0.5 |
| 30-34 * 2016 | -1.5 | -3.6, 0.74 | 0.2 |
| 25-29 * 2017 | -1.7 | -5.0, 0.94 | 0.2 |
| 30-34 * 2017 | -2.2 | -5.5, 0.47 | 0.13 |
| 25-29 * 2018 | -0.36 | -2.3, 1.7 | 0.7 |
| 30-34 * 2018 | -0.97 | -2.9, 1.1 | 0.3 |
| *^1^* aOR = adjusted Odds Ratio, CI = Confidence Interval | | | |
